# Face-to-Face Mentoring, Remotely Supervised Home Exercise Prehabilitation to Improve Physical Function in Patients Awaiting Kidney Transplantation: A Randomized Clinical Trial

**DOI:** 10.3389/fpsyg.2022.831445

**Published:** 2022-06-16

**Authors:** Xiaojie Ma, Zaozhang Zhang, Mengsi Peng, Bonuan Yao, Hongtao Jiang, Xuanfu Ji, Yong You

**Affiliations:** ^1^Department of Rehabilitation Medicine, The Second Affiliated Hospital of Hainan Medical University, Haikou, China; ^2^Department of General Practice, The Second Hospital of Hainan Medical University, Haikou, China; ^3^Department of Organ Transplantation, The Second Affiliated Hospital of Hainan Medical University, Haikou, China; ^4^Department of Rehabilitation Therapy Program, Hainan Medical University, Haikou, China; ^5^Department of Neurology, The Second Hospital of Hainan Medical University, Haikou, China

**Keywords:** end-stage renal disease, kidney transplantation, home exercise prehabilitation, physical fitness, cardiorespiratory fitness

## Abstract

**Objective:**

This study aims to explore the feasibility, safety, and effectiveness of home exercise prehabilitation on a new social platform for remote guidance to optimize the physical function of patients with end-stage renal disease awaiting kidney transplantation and provide scientific guidance on home prehabilitation exercises for patients awaiting kidney transplantation.

**Methods:**

The subjects of this randomized clinical trial were randomly divided into the test and control groups. The control group maintained their exercise habits, while the trial group was given a 12-week personalized home prehabilitation exercise prescription (aerobic exercise + functional resistance exercise + flexibility exercise) on a new social platform with remote guidance. The participants’ physical and cardiorespiratory fitness, quality of life, and psychological functioning were assessed before and after the intervention. The 6-min walk test (6MWT) walking distance and its percentage of attainment, the handgrip, the 5 repetition-sit-to-stand test, and the 4-m gait speed were used as primary outcome indicators, while the Short Form Health Survey SF-36 (health survey summary table) and the Hospital Anxiety and Depression scale were used as the secondary outcome indicators.

**Results:**

After 12 weeks of intervention, the changes in the 6MWT measured distance (+ 44.9 ± 40.2, *P* = 0.001) and the percentage of 6MWT measured distance achieved (+ 6.8 ± 5.7, *P* = 0.001), the handgrip (+ 2.7 ± 4.3, *P* = 0.028), the 5-sit-to-stand test (−1.1 ± 1.4, *P* = 0.005), and the 4-m walking speed (−0.3 ± 0.4, *P* < 0.001) of the test group (*n* = 21) improved significantly. In the control group (*n* = 16), the changes in the 6MWT measured distance (−13.1 ± 57.2), the 6MWT measured distance attainment percentage (−2.1 ± 9.1), the handgrip (−0.1 ± 2.5), the 5-sit-to-stand test value (0.6 ± 2.2), and the 4-m walking speed (0.2 ± 0.5) showed no significant difference. No significant improvement in anxiety, depression, and SF-36 was noted in both the test and control groups.

**Conclusion:**

The remote coaching of home exercise pre-habilitation on a new social platform significantly improves the physical and cardiopulmonary fitness of patients with end-stage renal disease awaiting kidney transplantation. This treatment is safe and feasible in this population.

## Introduction

End-stage renal disease (ESRD) refers to the development of the chronic kidney disease to the end stage in which the glomerular filtration rate is less than 15 mL/(min⋅1.73 m^2^). According to the World Health Organization, the incidence of chronic kidney disease in China exceeds 10.8%, and more than 0.03% of the patients develop ESRD (Zhang L. et al., 2012). ESRD seriously affects the life expectancy and quality of life of patients. The treatment of ESRD includes dialysis and kidney transplantation (Jha et al., 2013). Compared with dialysis treatment, kidney transplantation can achieve higher survival rates and quality of life, and lead to better clinical outcomes (Landreneau et al., 2010).

Due to the CKD stage and the dialysis treatment itself, ESRD patients often experience a decline in physical function while waiting for kidney transplantation. Previous research has reported that frailty and decreased cardiopulmonary function can lead to increased mortality and graft loss after kidney transplantation, placing a burden on Medicare (Bao et al., 2012). Previous research has shown ESRD patients are three times more likely to experience frailty than healthy persons (Lam and Jassal, 2015). Major surgeries, including the kidney transplantation, pose significant physiologic stress. Frailty and cardiopulmonary fitness are strongly associated with environmental and functional imbalances during the perioperative period. Frailty and decreased cardiopulmonary function increased rate of complications, such as delirium, delayed graft function, long hospital stay, increased early postoperative readmission rates, immunosuppressive intolerance, decreased quality of life, and increased mortality (Haugen et al., 2019). The cardiopulmonary function of the patients awaiting kidney transplantation was significantly low. In 56% of these patients awaiting kidney transplantation, the peak oxygen consumption was below 40% of the age-predicted value (Ting et al., 2014). Low peak oxygen consumption is associated with intensive care unit occupancy and mortality rate of patients undergoing kidney transplantation (Ting et al., 2013). Cardiovascular disease is the leading cause of death in patients with chronic kidney disease (Methven et al., 2017).

In patients undergoing non-emergency surgery, a good preoperative physical function implies few postoperative complications (Gillis et al., 2014). Prehabilitation is an exercise-based treatment program designed to improve the patient’s preoperative health for a better postoperative outcome (Cabilan et al., 2015). Lorenz et al. (2020) showed that multimodal exercise consisting of endurance training, strength training, and flexibility training twice a week for a total of 8 weeks while waiting for kidney transplantation can significantly improve fatigue, physical function, walking time, and grip strength. Manfredini et al. (2017) conducted a 6-month personalized home walking exercise program in 227 hemodialysis patients. The results showed that the experimental group increased the walking distance of the 6-minute walk test by 48 meters, and the time of 5-sit-to-stand test was reduced by 2.3 seconds. ESRD patients awaiting kidney transplantation are well suited for a prehabilitation program due to their functional decline and desire for kidney transplantation with expectations of improved physical health for increased probability of transplant success and reduced postoperative complication rates. Since dialysis and rehabilitation training cannot always be completed in the same medical facility, outpatient rehabilitation training increases the cost of transportation and time for patients. Some patients also require caregivers to accompany them, reducing the accessibility of outpatient rehabilitation. Home exercise prehabilitation can reduce the time and financial burden associated with regular visits to outpatient rehabilitation clinics. Unsupervised prehabilitation exercise programs at home for patients who underwent cardiac-related surgery (Waite et al., 2017) and with end-stage liver disease awaiting liver transplantation have been reported (Williams et al., 2019). However, there are fewer studies on patients awaiting kidney transplantation for prehabilitation. We hypothesize that home exercise prehabilitation through remote coaching and monitoring on the WeChat social platform and exercise bracelets could optimize the function of ESRD patients and optimize transplantation outcomes.

By evaluating the effects of home prehabilitation exercises on the physical and psychological functions of ESRD patients before kidney transplantation, we explore the feasibility of home exercise prehabilitation to optimize the physical functions of ESRD patients and provide a scientific and valuable guideline basis for home prehabilitation exercises for patients waiting for kidney transplantation.

## Materials and Methods

### Sample Size Calculation

The sample size calculation was based on the results of Rossi et al. (2014). The study is powered for the primary outcome measure of the improvement of exercise on the 6-minute walk test. Using the G-power software, the parameter values were set (i.e., effect size = 0.90, α = 0.05, and power = 0.80), the independent sample t-test was selected, the sample size was calculated as 32 people, and the expected shedding rate was 20%. Therefore, 40 subjects were actually needed for this experiment.

### Inclusion Criteria

The inclusion criteria were set as follows:

(i) Patients with end-stage renal disease (ESRD) estimated to have received their first kidney transplant at ≥ 12 weeks who were on the kidney transplant waiting list at the Second Affiliated Hospital of Hainan Medical College.(ii) the patients should be 18–60 years old;(iii) the patients volunteered and signed the informed consent form.

### Exclusion Criteria

The following patients were excluded from the study: (i) patients with unstable angina pectoris, new arrhythmia, recent myocardial infarction, or unstable cardiovascular events; (ii) patients who had a recent cerebrovascular accident or with a modified Rankin score (Modified Rankin Scale, mRS ≥ 3) (Yuan et al., 2012); and (iii) those who could not participate in the exercise due to other serious diseases.

### Subjects Recruitment

Sixty-seven subjects with ESRD were recruited in the Second Affiliated Hospital of Hainan Medical College from August 2020 to December 2020 among the patients who met the criteria for admission and awaiting kidney transplantation. Twelve of the 67 patients were excluded because they did not meet the inclusion criteria or satisfied an exclusion criterion, refused to participate, or could not use smartphones and sports bracelets.55 patients were randomly assigned in two groups: Control group (*n* = 27) and Test group (*n* = 28;12-week home exercise prehabilitation) Before the trial, all subjects were introduced to the trial process and signed an informed consent form. The trial protocol was approved by the Ethics Committee of the Second Affiliated Hospital of Hainan Medical College and registered with the China Clinical Trials Registry with registration number ChiCTR2000037846.

### Study Intervention

Specific details of the study design and methods have been published (Ma et al., 2021). Here, we report a subset of the data from the preoperative phase. This phase study was a randomized controlled design for 12 weeks. Patients with ESRD who are classified as candidates for kidney transplantation were evaluated and treated to explore the safety and feasibility of home exercise prehabilitation. Throughout the trial, all the subjects were asked to maintain their original dietary habits. The general information includes age, gender, type of occupation, marital status, education, body mass index (BMI), duration and frequency of dialysis, causes of ESRD, types of renal replacement therapy, and adverse effects. The cardiopulmonary function is evaluated by the 6MWT, and the maximum distance that the subjects can reach is measured. The 6MWT is significantly correlated with the peak oxygen consumption (ATS Committee on Proficiency Standards for Clinical Pulmonary Function Laboratories, 2002), which has good reliability and validity and can predict the adverse outcome of ESRD (Kohl et al., 2012). Physical fitness is evaluated by the handgrip, the 4-m gait speed, and the 5-sit-to-stand test (5R-STS). The grip and lower limb muscle strengths are good indicators of the systemic muscle strength (Ma, 2019) and reliable predictors of the increased risk of hospitalization and death (Guralnik et al., 2000). The quality of life is evaluated by the SF-36, which includes physical function, the limitation caused by the body, body pain, general health, vitality, social function, the limitation caused by emotion, and mental health. The higher the comprehensive score is, the better the quality of life is (Zhang Y. et al., 2012). The Hospital Anxiety and Depression scale will be used to evaluate the emotional state of anxiety and depression. Several anxiety and depression problems exist. The higher the total score is, the more serious the anxiety or depression is. This scale has good reliability and validity in our general hospitals, with anxiety Cronbach α = 0.76, and depression Cronbach α = 0.78 (Zheng et al., 2003).

#### Control Group

Renal replacement therapy, such as peritoneal dialysis or hemodialysis, was continued while waiting for kidney transplantation. Original exercise habits were also maintained. When the patients returned to the hospital after 12 weeks, they were re-evaluated for indicators, such as physical fitness and cardiopulmonary function.

#### Test Group

In addition to continuing their dialysis treatment while awaiting renal transplantation, individualized exercise prescriptions, including aerobic exercise, functional resistance training, and post-exercise session stretching, were developed and demonstrated for each patient by the same physical therapist on the basis of the functional assessment results. Supplement shows the detailed strategy of the exercise program. Patients were taught to apply the self-perceived exertion method (Borg6-20) to monitor the training intensity (Foster et al., 2001).

A WeChat group was established for the subjects. Home exercise diaries and exercise bracelets were distributed, and training on their proper use was conducted. Then, the home prehabilitation exercise was started. The exercise bracelet had to be turned on during the exercise to record the change in the exercise heart rate and the calories consumed. The heart rate, the calories consumed, photos or videos of the exercise process were uploaded to the WeChat group to “punch the card.” In addition to encouraging the patients to complete the exercise and clock in on time in the WeChat group, the experimenter conducted weekly telephone surveys after the start of the trial to encourage the patients to complete a home exercise diary and report any problems they encountered during their exercise, such as pain or discomfort, for the exercise prescription to be adjusted appropriately. The functional capacity, quality of life, and the emotional state of anxiety and depression of all the participants were reassessed by the same physical therapist after 12 weeks.

(1) Aerobic exercise: Individualized aerobic exercise prescriptions were developed for the subjects based on the results of the patient’s 6MWT assessment, including a 5-min warm up (slow walk), a 30-min moderate intensity walk (Ma, 2019), a 60%-of-heart-rate reserve walk, and a 5-min cool down (slow walk) (Supplementary Table 1).

(2) Functional resistance training: According to the weakness of the lower limbs (Zanotto et al., 2020) of the ESRD patients and the damage of the external oblique muscle, the internal oblique muscle, the transverse muscle, and the rectus abdominis muscle caused by Rutherford Morison and Alexandra incisions in the common operation methods of kidney transplantation, the following functional training was designed to strengthen the muscle strength reserve (Supplementary Table 2). The training movements include heel up, kick, squat up, dilatation of chest, bridge movement, and side pushing. This functional training is combined with breathing training, that is, exhale when exerting and inhale when relaxing, with each action repeated 8–12 times. The training intensity is defined by the self-perceived exertion (Borg11-13), and the frequency is 3 times a week.

(3) Post-exercise session stretching: After the end of the aerobic exercise and the resistance training, stretching is carried out for each resistance training muscle group (Supplementary Table 3). The training movements include stretching the front of the leg, stretching the back of the leg, and calf stretch. The duration of each action is 20 s, and each action is repeated 2–3 times, with a total time of approximately 10 min. Even breathing is maintained during the entire course.

### Statistical Analysis

The test data were analyzed using the SPSS24.0 statistical software with Office Excel 2016. The quantitative data were subjected to the Kolmogorov–Smirnov test to determine whether they followed a normal distribution. If the quantitative data followed a normal distribution, paired t tests were performed for within-group comparisons from baseline to the end of the study. Between-group differences at baseline and in the change from baseline to the end of the study were tested with unpaired t tests. If the data did not meet the normal distribution, then the Mann–Whitney U test was used for analysis. The results are expressed as mean ± standard deviation ($\bar{X}\pm S$). The greater the 6MWT distance and its percentage of achievement, the handgrip, and the quality of life were, the better, and the smaller the 4-m walking speed and the 5R-STS time were, the better. The change values were calculated according to the results of the two assessments, and change value = posttest value - initial value. Qualitative information was tested by the chi-square test (χ2 test), and the results are expressed as percentages (%). The significance level was *P* < 0.05. Given the statistical difference in the age comparison in the baseline data, age was used as an analysis of covariance to explore whether the age factor had an effect on each target variable.

## Results

**FIGURE 1:**
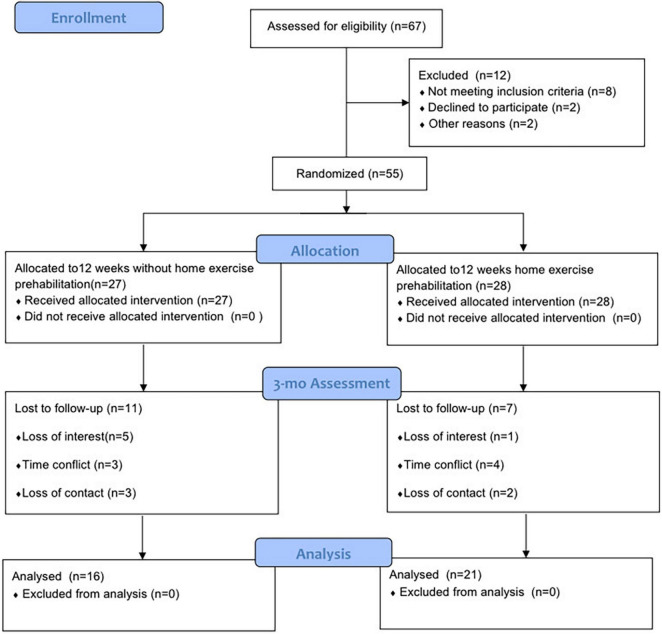
Flow chart of the test. 6MWT: 6-min walk test, 5R-STS: 5 repetition-Sit-to-stand test, SF-36: Short Form Health Survey SF-36.

During the intervention, 18 dropped out halfway through. Of the subjects who dropped, 11 and 7 belonged to the control and experimental groups, respectively. We did not assess post-intervention outcomes of the subjects who dropped out, so only 37 individuals were included in the outcome analysis. The flow of subjects is detailed in Figure 1, including the detailed reasons for exclusion and withdrawal.

### Basic Information

The mean age of the 37 patients was 41 years, and 67% were male. No significant differences were observed between the intervention and control groups in gender, BMI, occupation, marital status, education, type of renal replacement therapy, history of dialysis, history of previous comorbidities, and exercise habits (*P* > 0.05). A significant difference in age was noted between the two groups (*P* = 0.038). See Table 1 for details.

**TABLE 1:** Comparison of general conditions of subjects in both groups ($\bar{X}\pm S$).

	Test group (*n* = 21)	Control group (*n* = 16)	χ*^2^/t/Z*	*p*
Sex - Male (%)	14(66.7)	11(68.8)	0.018	1.000^†^
Age (years)	38.9 ± 10.5	45.9 ± 8.9	−2.153	0.038^#^
Height (cm)	164.4 ± 10.2	164.8 ± 5.0	−0.116	0.908^#^
Body weight (kg)	57.4 ± 11.3	59.2 ± 9.6	−0.517	0.608^#^
BMI (kg*m^–2^)	21.2 ± 3.5	21.8 ± 3.6	−0.555	0.582^#^
**Marital status (%)**				
Married	15(71.4)	15(81.1)	2.806	0.270^†^
Unmarried	5(23.8)	1(6.3)		
Divorced/widowed	1(4.8)	0(0)		
**Occupation(%)**				
Yes	11(52.4)	9(56.3)	1.629	0.877^†^
No	8(38.1)	5(31.3)		
Student	1(4.8)	0(0)		
Retired	1(4.8)	2(12.5)		
**Education level(%)**				
Undergraduate	5(23.8)	0(0)	7.520	0.081^†^
Specialized	5(23.8)	4(25)		
High School	1(4.8)	3(18.8)		
Junior High School	10(47.6)	7(43.8)		
Elementary School	0(0)	2(12.5)		
**Type of dialysis(%)**				
Hemodialysis	15(71.4)	11(68.8)	1.327	0.691^†^
Peritoneal dialysis	6(28.6)	4(25)		
Peritoneal dialysis + hemodialysis	0(0)	0(0)		
Did not start dialysis	0(0)	1(6.3)		
Dialysis history (years)	2.1 ± 2.2	4.6 ± 7.4	1.188	0.241*
Hypertension (%)	19(90.5)	16(100)	1.611	0.495^†^
Diabetes mellitus(%)	2(9.5)	1(6.3)	0.131	1.000^†^

BMI: Body Mass Index, Weight (kg)/height (m) 2.^†^Cardsquare test was used,^#^ Independent samples t-test was used, * Mann–Whitney U test was used.

### Primary Indicators

#### 6MWT Measured Distance

**FIGURE 2:**
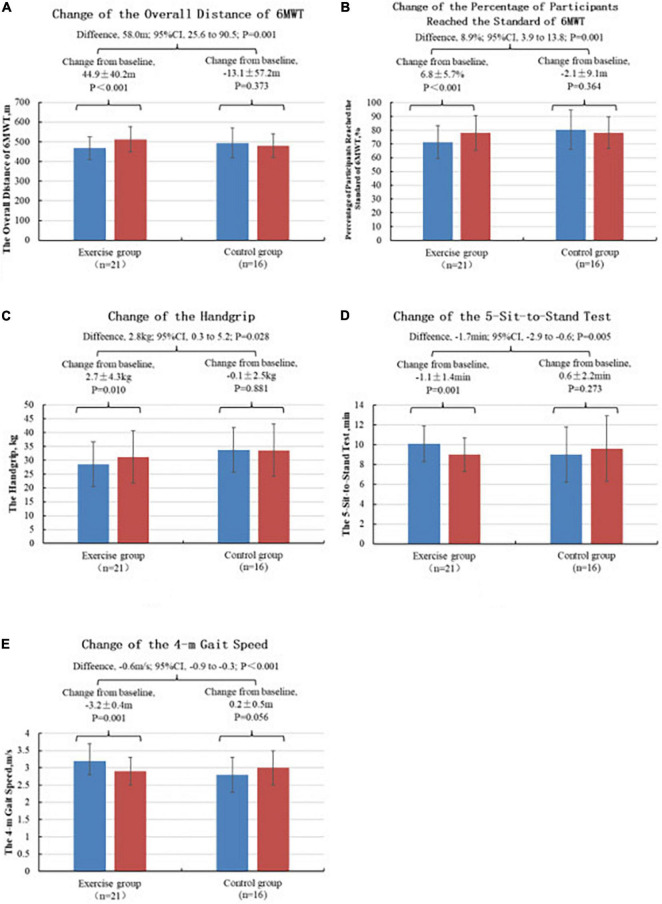
Changes in primary outcomes. **(A)** Comparison of the measured distance change values of 6MWT between two groups of subjects, ***P < 0.001. **(B)** Comparative analysis of the values of the percentage change in the measured distance attained for 6MWT in two groups of subjects, ***P < 0.001. **(C)** Comparative analysis of grip strength change values between the two groups of subjects, *P < 0.05. **(D)** Comparative analysis of the change values of the 5R-STS for the two groups of subjects, **P < 0.01. **(E)** Comparative analysis of the change values of the 4-meter gait speed for the two groups of subjects, ***P < 0.001. *p < 0.05, **p < 0.01, and ***p < 0.001.

Before the intervention, no significant difference in the measured 6MWT distance existed between the two groups (*P* > 0.05). No linear relationship existed between the age factor and the 6MWT measured distance, and the age factor had no significant effect on the 6MWT measured distance after treatment. After the intervention, a significant improvement in the measured 6-mindistance (*t* = −5.122, *P* < 0.001) was observed in the test group, whereas that in the control group decreased. The details are shown in Table 2. The values of the change in the 6MWT measured distance (difference between posttest and initial measurement) were calculated for both groups. The comparisons using the Mann–Whitney U test reveal a statistically significant improvement in the test group over the control group (*t* = −3.174, *P* = 0.001), as shown in detail in Figure 2A.

**TABLE 2:** Changes in primary outcomes.

	Test group (*n* = 21)	Control group (*n* = 16)	Between-group difference, Mean (95%CI)	Z/t	p	Effect size Cohen’s D
**6MWT distances**						
Pre-intervention	467.0 ± 58.6	493.6 ± 75.6	−26.6(−71.3∼18.2)	−1.206	0.236#	
Post-intervention	511.9 ± 64.5	480.4 ± 59.8	31.5(−10.7∼73.6)	−1.503	0.138*	0.506
Post-intervention-Pre-intervention	44.9 ± 40.2	−13.1 ± 57.2	58.0(25.6∼90.5)	−3.174	0.001*	1.173
**6MWT measured distance compliance percentage**						
Pre-intervention	71.3 ± 12.0	80.3 ± 14.1	−9.0(−17.7∼−0.2)	−2.087	0.044#	
Post-intervention	78.0 ± 12.7	78.1 ± 11.5	−0.1(−8.3∼8.1)	0.491	0.639*	−0.008
Post-intervention-Pre-intervention	6.8 ± 5.7	−2.1 ± 9.1	8.9(3.9∼13.8)	−3.097	0.001*	1.172
**Grip strength**						
Pre-intervention	28.6 ± 8.1	33.7 ± 8.1	−5.2(−10.6∼0.3)	−1.929	0.062#	
Post-intervention	31.2 ± 9.4	33.6 ± 9.5	−2.4(−8.8∼4.0)	−0.764	0.450#	−0.254
Post-intervention-Pre-intervention	2.7 ± 4.3	−0.1 ± 2.5	2.8(0.3∼5.2)	2.293	0.028#	0.796
**5-sit-to-stand test (5R-STS)**						
Pre-intervention	10.1 ± 1.8	9.0 ± 2.8	1.1(−0.4∼2.7)	1.500	0.143#	
Post-intervention	9.0 ± 1.7	9.6 ± 3.3	−0.6(−2.3∼1.1)	0.123	0.916*	−0.229
Post-intervention-Pre-intervention	−1.1 ± 1.4	0.6 ± 2.2	−1.7(−2.9∼−0.6)	−2.976	0.005#	−0.922
**4-m gait speed**						
Pre-intervention	3.2 ± 0.5	2.8 ± 0.5	0.4(0.1∼0.7)	2.377	0.023#	
Post-intervention	2.9 ± 0.4	3.0 ± 0.5	−0.2(−0.5∼0.1)	−1.372	0.179#	−0.221
Post-intervention-Pre-intervention	−0.3 ± 0.4	0.2 ± 0.5	−0.6(−0.9∼−0.3)	−4.154	<0.001#	0.221

6MWT: 6-minute walking test, CI: confidence interval.* Mann–Whitney U test was used, ^#^ independent samples t test was used.

#### 6MWT Measured Distance Compliance Percentage

Before the intervention, the percentage of the 6MWT measured distance achieved was higher in the control group than in the test group, with a significant difference of *P* = 0.044. No linear relationship existed between the age factor and the percentage of the 6MWT measured distance, and the age factor had no significant effect on the percentage of the 6MWT measured distance achieved after the treatment. After the intervention, the percentage of the 6-min walk measured distance achieved (*t* = −5.437, *P* < 0.001) exhibited significant improvement in the test group and no improvement in the control group. The details are shown in Table 2. The value of the percentage change in the 6MWT measured distance achieved (difference between the posttest and the initial measurement) was calculated for both groups. The comparisons using the Mann–Whitney U test reveal a statistically significant improvement in the test group compared with the control group (*t* = −3.097, *p* = 0.001). Figure 2B presents the details.

#### Grip Strength

Before the intervention, no significant difference in grip strength existed between the two groups (*P* > 0.05). No linear relationship existed between the age factor and the grip strength, and the age factor had no significant effect on the grip strength after the treatment. After the intervention, a significant improvement in grip strength (*t* = −2.844, *P* < 0.010) was observed in the test group, whereas that in the control group decreased. The details are shown in Table 2. The changes in the grip strength values (difference between post- and initial tests) were calculated for both groups and compared using the independent samples t-test, revealing that the improvement in the test group was significantly greater than that in the control group (*t* = 2.293, *P* = 0.028), and the difference was statistically significant. See Figure 2C for details.

#### 5-Sit-to-Stand Test (5R-STS)

Before the intervention, no significant difference existed between the two groups in terms of the time 5R-STS (*p* > 0.05). After the intervention, a significant improvement in 5R-STS (*t* = 3.737, *P* = 0.001) in the test group was achieved after adjusting the age factor. Table 2 presents the details. The calculation of the values of the change in 5R-STS (difference between the posttest and the initial test) between the two groups using an independent samples *t*-test shows that the improvement in the test group was significantly greater than in the control group (*t* = −2.976, *P* = 0.005), and the difference was statistically significant. Figure 2D presents the details.

#### 4-m Gait Speed

Before the intervention, the 4-m gait speed in the control group was higher than that than in the test group, with a significant difference of *p* = 0.023. No linear relationship existed between the age factor and the 4-m gait speed, and the age factor had no significant effect on the 4-m gait speed after treatment. The smaller the indicator of the 4-m gait speed was, the better. A significant improvement in 4-m gait speed was observed in the test group (*t* = 4.040, *p* = 0.001) after the intervention, whereas that in the control group decreased. Table 2 presents the details. The value of the change in the 4-m gait speed (difference between the posttest and the initial test) was calculated for both groups and compared using an independent samples *t*-test, and the improvement was found to be significantly greater in the test group than in the control group (*t* = −4.154, *P* < 0.001), with a statistically significant difference. Figure 2E presents the details.

### Secondary Outcomes

#### Anxiety and Depression

Before the intervention, no significant difference in anxiety and depression existed between the two groups (*P* > 0.05). After the intervention, no statistically significant difference existed between the anxiety and depression in the test and control groups. The change values (the difference between the posttest and the initial test) of the two groups were calculated and compared using the Mann–Whitney U test, revealing that no statistically significant difference existed between the change values of anxiety and depression in the test and control groups. Table 3 presents the details.

**TABLE 3:** Changes in secondary outcomes.

	Test group (*n* = 21)	Control group (*n* = 16)	Between-group difference, Mean (95%CI)	*Z/t*	*p*	effect size Cohen’s D
**Anxiety**						
Pre-intervention	4.6 ± 2.2	4.7 ± 3.7	1.0(−2.1–1.9)	−0.324	0.751*	
Post-intervention	4.1 ± 3.7	5.1 ± 3.7	−1.0(−3.55–1.5)	−0.806	0.426^#^	−0.270
Post-intervention-Pre-intervention	−0.5 ± 2.6	0.4 ± 2.2	0.8(−2.6–0.7)	1.731	0.089*	−0.374
**Depression**						
Pre-intervention	4.8 ± 3.0	5.1 ± 3.9	−0.3(−2.6–2.0)	0.201	0.844*	
Post-intervention	5.2 ± 3.2	5.4 ± 4.1	−0.2(−2.7–2.2)	−0.204	0.839^#^	−0.027
Post-intervention-Pre-intervention	0.4 ± 3.4	0.4 ± 3.2	0.1(−2.2–2.3)	0.372	0.728*	0
**SF-36**						
Pre-intervention	112.4 ± 9.7	112.3 ± 14.8	0.1(−8.0–8.3)	0.069	0.972^#^	
Post-intervention	112.7 ± 13.7	111.8 ± 16.2	1.0(−9.0–10.9)	0.195	0.847^#^	0.060
Post-intervention-Pre-intervention	0.3 ± 11.3	−0.5 ± 11.2	0.8(−6.8–8.4)	−0.015	0.988*	0.071

SF-36: Short Form Health Survey SF-36,CI: confidence interval.* Mann–Whitney U test was used, ^#^ independent samples t test was used; ^a^ paired samples t test was used.

According to the HADS score scale of 0–7 normal, 8–10 mild depression/anxiety, 11–14 moderate, and 15–21 severe, the patients who satisfied the anxiety-depression status (≥ 7 points) prior to the intervention were compared. Before the intervention, no significant difference in anxiety and depression existed between the two groups (*p* > 0.05). After the intervention, the anxiety in both groups improved, with no significant difference (*P* = 0.411). Depression increased in both groups but no significant difference was noted. The change values (difference between the posttest and the initial test) were calculated for both groups and compared using the Mann–Whitney U test, and no statistically significant differences were found between the anxiety and depression change values of the test and control groups(Supplementary Table 4 in Supplement).

#### Quality of Life

Before the intervention, no significant difference in SF-36 existed between the two groups (*P* > 0.05). The greater the index of quality of life was, the better. After the intervention, the quality of life in the test group improved, but no significant difference was observed. The quality of life of the control group decreased, with no significant difference. The SF-36 change values (the difference between the posttest and the initial test) were calculated for both groups and compared using the Mann–Whitney U test, and no statistically significant differences were found between the SF-36 change values of the test and control groups (Table 3).

## Discussion

The high incidence of ESRD severely affects the life expectancy and quality of life of patients. Kidney transplantation can improve the survival and quality of life of ESRD patients. However, the cardiopulmonary function debilitates and declines in ESRD patients awaiting kidney transplantation. In the present study, the average 6MWT distance measured in 37 subjects only reached 75% of the normal predicted values. The decrease in function can lead to increased mortality and graft loss after kidney transplantation (Bao et al., 2012). Manfredini study showed that exercise therapy during dialysis increases the maximal oxygen uptake and exercise duration in ESRD patients (Methven et al., 2017). In the study of Mara on the preoperative prehabilitation for kidney transplantation, weekly outpatient prehabilitation for 3–6 months before surgery was found to significantly improve ESRD patients’ mobility (McAdams-DeMarco et al., 2019). However, regular outpatient exercise programs can impose time and financial burdens on patients with ESRD. This situation limits the widespread use of outpatient exercise in this patient population. Patients prefer home and community-based exercise to exercise during hemodialysis in the hospital (Sieverdes et al., 2015). The results of this study show that 12 weeks of home prehabilitation exercise on a new social platform with remote guidance improved the physical fitness (grip strength, 5R-STS, 4-m gait speed) and cardiorespiratory fitness (6-min walking distance and its percentage of attainment) of ESRD patients awaiting kidney transplantation. In contrast, the physical performance (grip strength, 5R-STS, 4-m gait speed) and the cardiopulmonary function (6-mine walking distance and its percentage of attainment) decreased after 12 weeks in the control group, which did not differ from the baseline physical performance and cardiopulmonary function levels of the test group. Twelve weeks of home prehabilitation exercises can indeed improve the physical fitness and cardiorespiratory function of ESRD patients waiting for kidney transplantation.

In a study of patients undergoing cardiac-related surgery (coronary artery bypass grafting and heart valve surgery), Waite found that preoperative home prehabilitation reduces debilitation, improves the physical function, and shortens the length of hospital stay (Waite et al., 2017). Williams also found that home exercise improves the aerobic capacity and functional status of patients awaiting liver transplantation (Williams et al., 2019). However, relevant reports on kidney transplant waiting patients are limited. Uchiyama et al. (2021) divided patients with Stage 4 CKD into exercise and control groups and found that 6 month home-based exercise program improved aerobic capacity and health related quality of life in patients with Stage 4 CKD.

The exercise in this study was performed unsupervised at home. To increase patient compliance, we educated the patients about the need for preoperative prehabilitation. We provided each participant with a home exercise diary and an exercise bracelet, and the exercise prescriptions were printed in pictures and text in the exercise diary. We also recorded videos of relevant exercise movements and sent them to the patients. We set up a WeChat group and daily exercise punch cards, provided encouragement within the group, and made weekly follow-up phone calls. After recruiting the subjects, 94% of those who met the eligibility criteria agreed to participate in this trial, indicating that ESRD patients have a good acceptance of home exercise prehabilitation, and 75% of these patients completed the full intervention. The 12-week home prehabilitation exercise for ESRD patients was confirmed to be feasible.

There was a significant difference in age between the two groups at baseline statistical analysis. Analysis of covariance was used to adjust for baseline age measurements. No linear relationship existed between the age factor and the outcomes. But we cannot exclude that it may raise some questions regarding the observed results. For example, it was precisely because the experimental group was younger that the physical function improved better after exercise. This needs to be verified in subsequent larger and more rigorous RCTs. One peritoneal dialysis subject in this study had symptoms of generalized edema, weight gain, and scrotal edema 2 months prior to entry into this study. The edema symptoms were exacerbated by scrotal edema during prehabilitation with home exercise. The above symptoms improved after intensive peritoneal dialysis ultrafiltration treatment, indicating that home exercise prehabilitation is safe.

Manfredini showed in a study on ESRD patients that a 6-month low-intensity walking program improved the depression and quality of life scores (Manfredini et al., 2017). In contrast, no significant improvement in the anxiety-depression and quality of life scores was observed in the intervention and control groups of this study. This may be related to the small sample size of this study, which has a short period of only 12 weeks, and to the fact that the subjects did not meet the diagnostic criteria for anxiety-depression scores at the baseline assessment for anxiety (4.6 ± 2.2) and depression (4.8 ± 3.0) scores.

This study is the first exploratory trial of a home prehabilitation campaign for ESRD patients awaiting kidney transplantation with a small sample size. This study was conducted in a single center, with limitations for future multi-center replication. In future studies, the real-time monitoring and collection of patient exercise records, the mastery of intensity, and increased communication and trust building between rehabilitation therapists and patients are issues that need further improvement. This study combined three types of exercise (aerobic exercise, strength training, and post-exercise session stretching). One of the exercises may have primarily affected the physical performance and the cardiorespiratory fitness. Future trials could further investigate the effects of the type and amount of exercise on the physical and cardiorespiratory fitness of this group of individuals. In addition, a larger sample size and multicenter randomized controlled trials are needed for validation in the future.

## Conclusion

The 12-week home exercise prehabilitation on a new social platform with remote guidance improves the physical and cardiorespiratory fitness of patients with ESRD awaiting renal transplantation, and unsupervised home exercise is safe, effective, and feasible in this population.

## Data Availability Statement

The original contributions presented in the study are included in the article/Supplementary Material, further inquiries can be directed to the corresponding author/s.

## Ethics Statement

The studies involving human participants were reviewed and approved by Ethics Committee of the Second Affiliated Hospital of Hainan Medical College. The patients/participants provided their written informed consent to participate in this study. The individual(s) provided their written informed consent for the publication of any identifiable images or data presented in this article.

## Author Contributions

XM, HJ, and YY led the project, established the protocol, drafted the initial manuscript, and reviewed and revised the manuscript. XM, ZZ, and MP wrote and revised the manuscript. MP, XJ, and BY managed and analyzed the data curation. YY and HJ wrote and reviewed the final version. All authors approved the final manuscript as submitted and agreed to be accountable for all aspects of the work.

## Conflict of Interest

The authors declare that the research was conducted in the absence of any commercial or financial relationships that could be construed as a potential conflict of interest.

## Publisher’s Note

All claims expressed in this article are solely those of the authors and do not necessarily represent those of their affiliated organizations, or those of the publisher, the editors and the reviewers. Any product that may be evaluated in this article, or claim that may be made by its manufacturer, is not guaranteed or endorsed by the publisher.

## References

[B1] ATS Committee on Proficiency Standards for Clinical Pulmonary Function Laboratories (2002). on Proficiency Standards for Clinical Pulmonary Function Laboratories. ATS statement: guidelines for the six-minute walk test. *Am. J. Respir. Crit. Care Med.* 166 111–117. 10.1164/ajrccm.166.1.at1102 12091180

[B2] Bao Y. Dalrymple L. Chertow G. M. Kaysen G. A. Johansen K. L. (2012). Frailty, dialysis initiation, and mortality in end-stage renal disease. *Arch. Intern. Med.* 172 1071–1077. 10.1001/archinternmed.2012.3020 22733312 PMC4117243

[B3] Cabilan C. J. Hines S. Munday J. (2015). The effectiveness of prehabilitation or preoperative exercise for surgical patients: a systematic review. *JBI Database system. Rev. Implement. Rep.* 13 146–187. 10.11124/jbisrir-2015-1885 26447015

[B4] Foster C. Florhaug J. A. Franklin J. Gottschall L. Hrovatin L. A. Parker S. (2001). A new approach to monitoring exercise training. *J. Strength Cond. Res.* 15 109–115. 10.1519/1533-4287(2001)015<0109:anatme>2.0.co;2 11708692

[B5] Gillis C. Li C. Lee L. Awasthi R. Augustin B. Gamsa A. (2014). Prehabilitation versus rehabilitation: a randomized control trial in patients undergoing colorectal resection for cancer. *Anesthesiology* 121 937–947. 10.1097/ALN.0000000000000393 25076007

[B6] Guralnik J. M. Ferrucci L. Pieper C. F. Leveille S. G. Markides K. S. Ostir G. V. (2000). Lower extremity function and subsequent disability: consistency across studies, predictive models, and value of gait speed alone compared with the short physical performance battery. *J. Gerontol. A Biol. Sci. Med. Sci.* 55 M221–M231. 10.1093/gerona/55.4.m221 10811152 PMC12149745

[B7] Haugen C. E. Chu N. M. Ying H. Warsame F. Holscher C. M. Desai N. M. (2019). Frailty and Access to Kidney Transplantation. *Clin. J. Am. Soc. Nephrol.* 14 576–582. 10.2215/CJN.12921118 30890577 PMC6450348

[B8] Jha V. Garcia-Garcia G. Iseki K. Li Z. Naicker S. Plattner B. (2013). Chronic kidney disease: global dimension and perspectives. *Lancet* 382 260–272. 10.1016/S0140-6736(13)60687-X 23727169

[B9] Kohl L. M. Signori L. U. Ribeiro R. A. Silva A. M. Moreira P. R. Dipp T. (2012). Prognostic value of the six-minute walk test in end-stage renal disease life expectancy: a prospective cohort study. *Clinics* 67 581–586. 10.6061/clinics/2012(06)06 22760895 PMC3370308

[B10] Lam M. Jassal S. V. (2015). The concept of frailty in geriatric chronic kidney disease (CKD) patients. *Blood purificat.* 39 50–54. 10.1159/000368952 25661193

[B11] Landreneau K. Lee K. Landreneau M. D. (2010). Quality of life in patients undergoing hemodialysis and kidney transplantation–a meta-analytic review. *Nephrol. Nurs. J* 37 37–44. 20333902

[B12] Lorenz E. C. Hickson L. J. Weatherly R. M. Thompson K. L. Walker H. A. Rasmussen J. M. (2020). Protocolized exercise improves frailty parameters and lower extremity impairment: A promising prehabilitation strategy for kidney transplant candidates. *Clin Transplant.* 34:e14017. 10.1111/ctr.14017 32573816 PMC7721982

[B13] Ma X. Zhang Z. Yao B. Peng M. Jiang H. You Y. (2021). Study on the effect of pre-rehabilitation home-based on patients undergoing kidney transplantation with end-stage renal disease: A study protocol. *Medicine* 100:e28280. 10.1097/MD.0000000000028280 34967359 PMC8718243

[B14] Ma Y. (2019). Kidney Rehabilitation Committee of the Rehabilitation Physicians Branch of the Chinese Medical Association. Expert consensus on exercise rehabilitation for adults with chronic kidney disease in China. *Chinese J. Nephrol.* 35 537–543. 10.3760/cma.j.issn.1001⋅7097.2019.07.011 30704229

[B15] Manfredini F. Mallamaci F. D’Arrigo G. Baggetta R. Bolignano D. Torino C. (2017). Exercise in Patients on Dialysis: A Multicenter, Randomized Clinical Trial. *J. Am. Soc. Nephrol.* 28 1259–1268. 10.1681/ASN.2016030378 27909047 PMC5373448

[B16] McAdams-DeMarco M. A. Ying H. Van Pilsum Rasmussen S. Schrack J. Haugen C. E. Chu N. M. (2019). Prehabilitation prior to kidney transplantation: Results from a pilot study. *Clin. Transplant.* 33:e13450. 10.1111/ctr.13450 30462375 PMC6342659

[B17] Methven S. Steenkamp R. Fraser S. U. K. (2017). UK Renal Registry 19th Annual Report: Chapter 5 Survival and Causes of Death in UK Adult Patients on Renal Replacement Therapy in 2015: National and Centre-specific Analyses. *Nephron* 137 117–150. 10.1159/000481367 28930724

[B18] Rossi A. P. Burris D. D. Lucas F. L. Crocker G. A. Wasserman J. C. (2014). Effects of a renal rehabilitation exercise program in patients with CKD: a randomized, controlled trial. *Clin. J. Am. Soc. Nephrol.* 9 2052–2058. 10.2215/CJN.11791113 25414318 PMC4255415

[B19] Sieverdes J. C. Raynor P. A. Armstrong T. Jenkins C. H. Sox L. R. Treiber F. A. (2015). Attitudes and perceptions of patients on the kidney transplant waiting list toward mobile health-delivered physical activity programs. *Prog. Transplant.* 25 26–34. 10.7182/pit2015884 25758797 PMC4751589

[B20] Ting S. M. Iqbal H. Hamborg T. Imray C. H. Hewins S. Banerjee P. (2013). Reduced functional measure of cardiovascular reserve predicts admission to critical care unit following kidney transplantation. *PLoS One* 8:e64335. 10.1371/journal.pone.0064335 23724043 PMC3664577

[B21] Ting S. M. Iqbal H. Kanji H. Hamborg T. Aldridge N. Krishnan N. (2014). Functional cardiovascular reserve predicts survival pre-kidney and post-kidney transplantation. *J. Am. Soc. Nephrol.* 25 187–195. 10.1681/ASN.2013040348 24231666 PMC3871777

[B22] Uchiyama K. Adachi K. Muraoka K. Nakayama T. Oshida T. Yasuda M. (2021). Home-based aerobic exercise and resistance training for severe chronic kidney disease: a randomized controlled trial. *J. Cachexia Sarcopenia Muscle* 12, 1789–1802. 10.1002/jcsm.12775 34554649 PMC8718025

[B23] Waite I. Deshpande R. Baghai M. Massey T. Wendler O. Greenwood S. (2017). Home-based preoperative rehabilitation (prehab) to improve physical function and reduce hospital length of stay for frail patients undergoing coronary artery bypass graft and valve surgery. *J. Cardiothorac. Surg.* 12:91. 10.1186/s13019-017-0655-8 29073924 PMC5658994

[B24] Williams F. R. Vallance A. Faulkner T. Towey J. Durman S. Kyte D. (2019). Home-Based Exercise in Patients Awaiting Liver Transplantation: A Feasibility Study. *Liver Transpl.* 25 995–1006. 10.1002/lt.25442 30859755

[B25] Yuan J. L. Bruno A. Li T. Li S. J. Zhang X. D. Li H. Y. (2012). Replication and extension of the simplified modified rankin scale in 150 Chinese stroke patients. *Eur. Neurol.* 67 206–210. 10.1159/000334849 22377778

[B26] Zanotto T. Gobbo S. Bullo V. Vendramin B. Roma E. Duregon F. (2020). Postural balance, muscle strength, and history of falls in end-stage renal disease patients living with a kidney transplant: A cross-sectional study. *Gait Posture.* 76 358–363. 10.1016/j.gaitpost.2019.12.031 31901763

[B27] Zhang L. Wang F. Wang L. Wang W. Liu B. Liu J. (2012). Prevalence of chronic kidney disease in China: a cross-sectional survey. *Lancet* 379 815–822. 10.1016/S0140-6736(12)60033-6 22386035

[B28] Zhang Y. Qu B. Lun S. S. Guo Y. Liu J. (2012). The 36-item short form health survey: reliability and validity in Chinese medical students. *Int. J. Med. Sci.* 9 521–526. 10.7150/ijms.4503 22991490 PMC3444972

[B29] Zheng L. Wang Y. Li H. (2003). Application of Hospital Anxiety and Depression Scale in general hospital: an analysis in reliability and validity. *Shanghai Arch. Psychiatr.* 264–266. 10.3969/j.issn.1002-0829.2003.05.003

