# Supplementary material for: Face-to-Face Mentoring, Remotely Supervised Home Exercise Prehabilitation to Improve Physical Function in Patients Awaiting Kidney Transplantation: A Randomized Clinical Trial
Source: Front Psychol. 2022 Jun 16;13:831445. doi: 10.3389/fpsyg.2022.831445 (PMC9245540; doi:10.3389/fpsyg.2022.831445)
Supplement: Supplementary file 1 [file Table_1.DOCX]

Supplementary Material

# Aerobic exercise:

Individualized aerobic exercise prescriptions were developed for the subjects on the basis of the results of the patient’s 6-min walk test (6MWT) assessment, including a 5-min warm up (slow walk), a 30-min moderate intensity (60% of heart rate reserve) walk, and a 5-min cool down (slow walk). Table 1 provides an overview of the aerobic exercise prescription.

**eTable 1. Aerobic exercise prescription**

| Type | Walk |
| --- | --- |
| Intensity | Moderate intensity (40%–60% of the heart rate reserve value) is calculated according to the results of the resting heart rate, the maximum heart rate, and the distance in the 6MWT. The walking distance is given to the subjects, and the self-perceived exertion method (Borg11–13) is used to monitor the intensity during exercise.  For example, in the 6MWT, the resting heart rate is 65 beats/min, the maximum heart rate is 95 beats/min, and the distance is 500 m.  Target heart rate: (maximum heart rate-resting heart rate) * 60% + resting heart rate = 83 (+/-5) beats/min, Borg fatigue score is 11–13, 500X60%X5 = 2125. A 30-min walk of 2,125 m is recommended for the patients. |
| Time | Walk continuously or intermittently for 40 min.  (including 5 min of warm up, 30 min of exercise, and 5 min of cool down) |
| Frequency | Five times a week |

# Functional resistance training：

According to the weakness of the lower limbs of ESRD patients and the damage of their external oblique, internal oblique, transverse, and rectus abdominis muscles caused by Rutherford Morison and Alexandra incisions in common operation methods of kidney transplantation, the following functional training was designed to strengthen the muscle strength reserve (eTable 2). This process is combined with breathing training, that is, exhale when exerting and inhale when relaxing, and each action is repeated 8–12 times. The training intensity is defined by the self-perceived exertion (Borg11–13), and the frequency is 3 times a week.

**eTable 2. Functional resistance training**

| Training action | Action diagram | Action points |
| --- | --- | --- |
| Heel up | 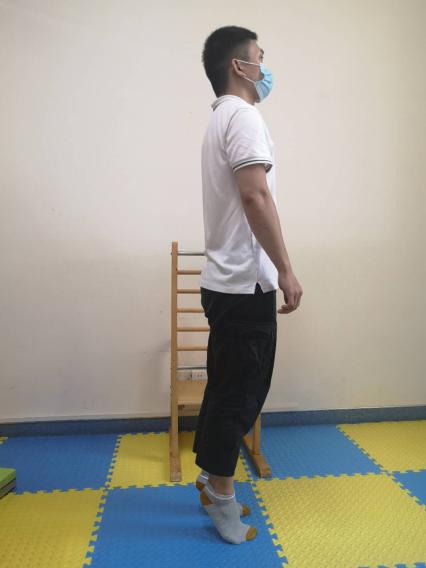 | Hold the object by hand, keep your legs as wide as your shoulders, exhale, and slowly lift your heels to the highest point to maintain 3–5 s (calm breathing here). Then, exhale and slowly land on your heels to relax. |
| Kick | 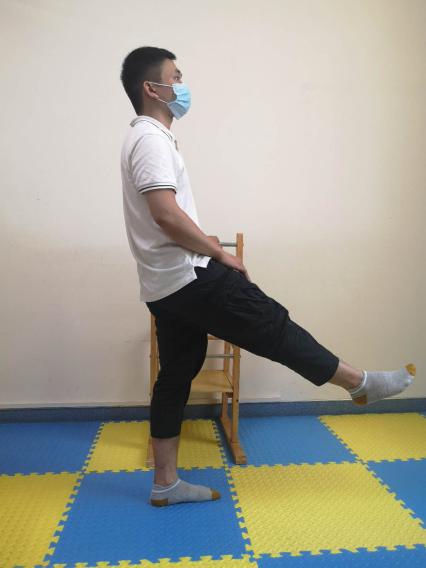 | Hold the object by hand, keep the legs as wide as the shoulder, and one side of the lower limb bend the hip and knees. Then, keep the hip flexion and straighten the knee, and then extend the entire lower limb. Repeat this action alternately from left to right. |
| Squat up | 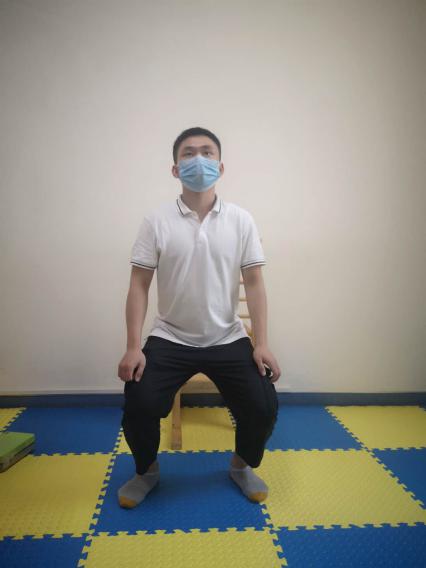 | Hold the object by hand, feet apart, shoulder wide, exhale with knee flexion not exceeding toe, the center of gravity sinking, maintain 3–5 s (calm breathing here), exhale and stand up and relax. |
| Dilatation of chest | 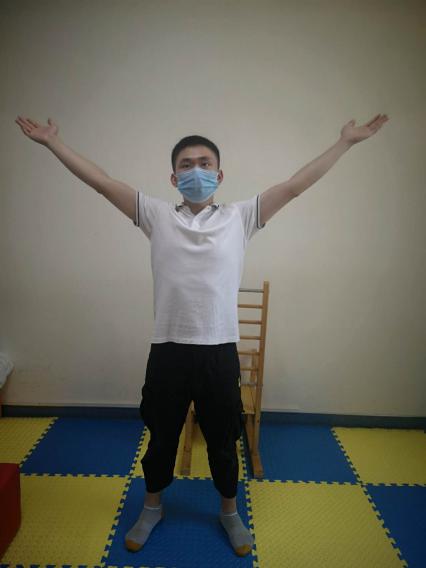 | Keep your arms straight, gradually raise your head forward and upward, and inhale deeply at the same time; then, retract your arms while exhaling deeply. |
| Bridge movement | 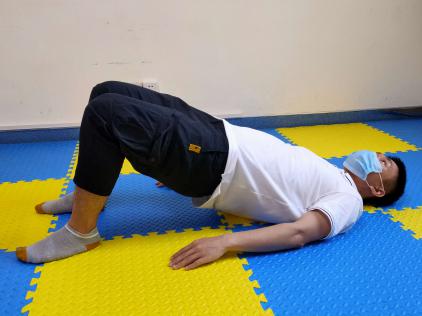 | Take a supine position, hands flat on both sides of the body, legs flexion, feet flat on the bed, arms lifted off the bed, maintain 3–5 s (calm breathing here), exhale, and relax. |
| side pushing | 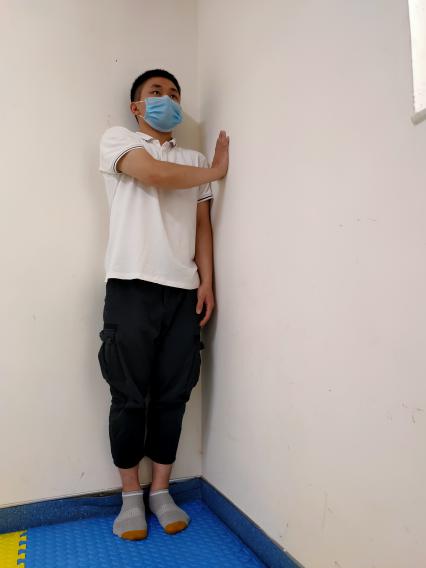 | Stand in the corner of the wall, back against one wall, push the other wall with the outside hand, maintain 3-5 s (breathe calmly here), relax and proceed alternately from side to side. |

# Post-exercise session stretching:

After the end of the aerobic exercise and the resistance training, stretching is performed for each resistance training muscle group (eTable 3). The duration of each action is 20 s, which is repeated 2–3 times, the total time is 10 min, and even breathing is maintained during the entire course.

**eTable 3. Post-exercise session stretching**

| Training action | Action diagram | Action points |
| --- | --- | --- |
| Stretch the front of the leg | 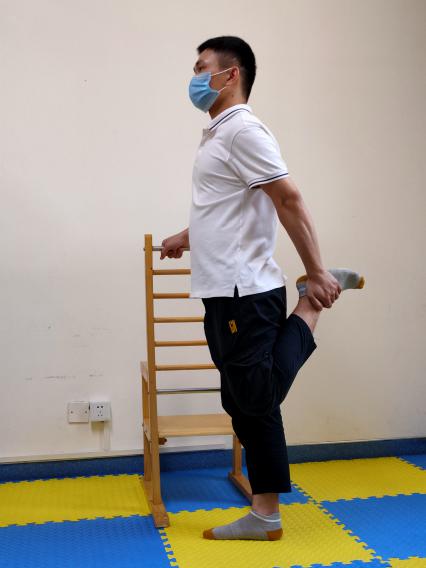 | Hold the object with your hands, stand on one leg, grab the left foot with your left hand, and pull it toward your buttocks. Keep the upper body as straight as possible (feet as close to the buttocks as possible) and maintain for 20 s, 30 s, left, and right alternately. |
| Stretch the back of the leg | 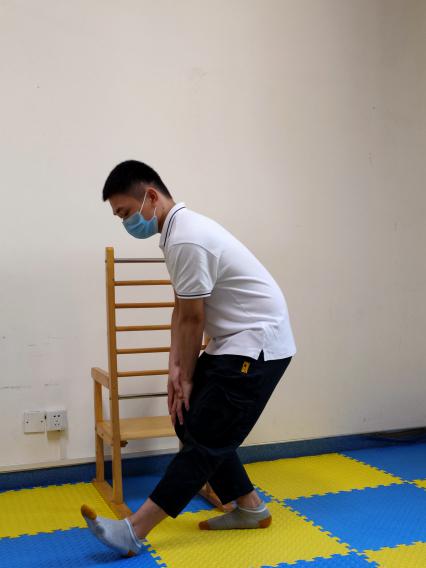 | Bend your right leg, hold your left knee with both hands, straighten your left leg and hook your toes, bend your waist and back straight down, and maintain the pose for 20 s-30 s, left and righ alternately. |
| Calf stretch | 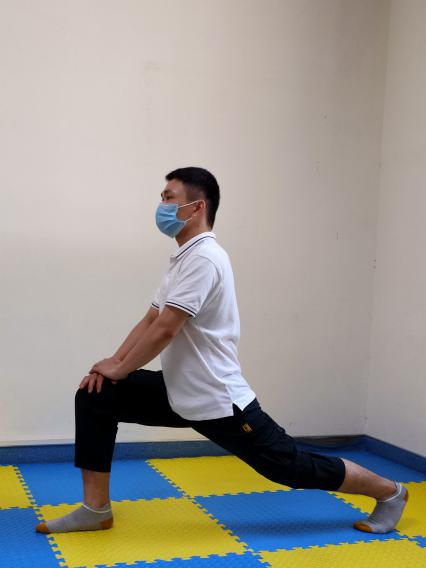 | The right leg in front of the bow and arrow, the left toe forward, the body does a forward, downward elastic stretch, and the left heel does not leave the ground. The total process takes 20–30 s. |

**eTable 4 Comparison of pre- and post-intervention scores between the two groups of patients eligible for anxiety**–**depression status**

|  | Test group  (*n* = 21) | Control group  (*n* = 16) | *Z/t* | *p* | Effect size  Cohen's D |
| --- | --- | --- | --- | --- | --- |
| **Anxiety** |  |  |  |  |  |
| Pre-intervention | 7.8(8.0) | 9.75(9.5) | -1.014 | 0.310 |  |
| Post-intervention | 7.4(6.0) | 8.75(9.0) | -0.615 | 0.539^#^ | -0.177 |
| *t* | 1.604 | 0.365 |  |  |  |
| *p^a^* | 0.109 | 0.715 |  |  |  |
| Post-intervention－Pre-intervention | -0.4(-2.0) | -1.0(-0.5) | -0.496 | 0.620^*^ | 0.412 |
| **Depression** |  |  |  |  |  |
| Pre-intervention | 6.0(7.0) | 7.5(8.0) | 0.876 | 0.381^*^ |  |
| Post-intervention | 8.0(6.0) | 8.75(8.5) | -0.738 | 0.461^#^ | -0.102 |
| *t* | 0.000 | 0.000 |  |  |  |
| *p^a^* | 1.000 | 1.000 |  |  |  |
| Post-intervention－Pre-intervention | 2.0（3.0） | 1.25（2.0） | -0.124 | 0.901^*^ | 0.294 |

CI: confidence interval.^*^ Mann-Whitney U test was used, ^#^ independent samples t test was used; ^a^ paired samples t test was used.


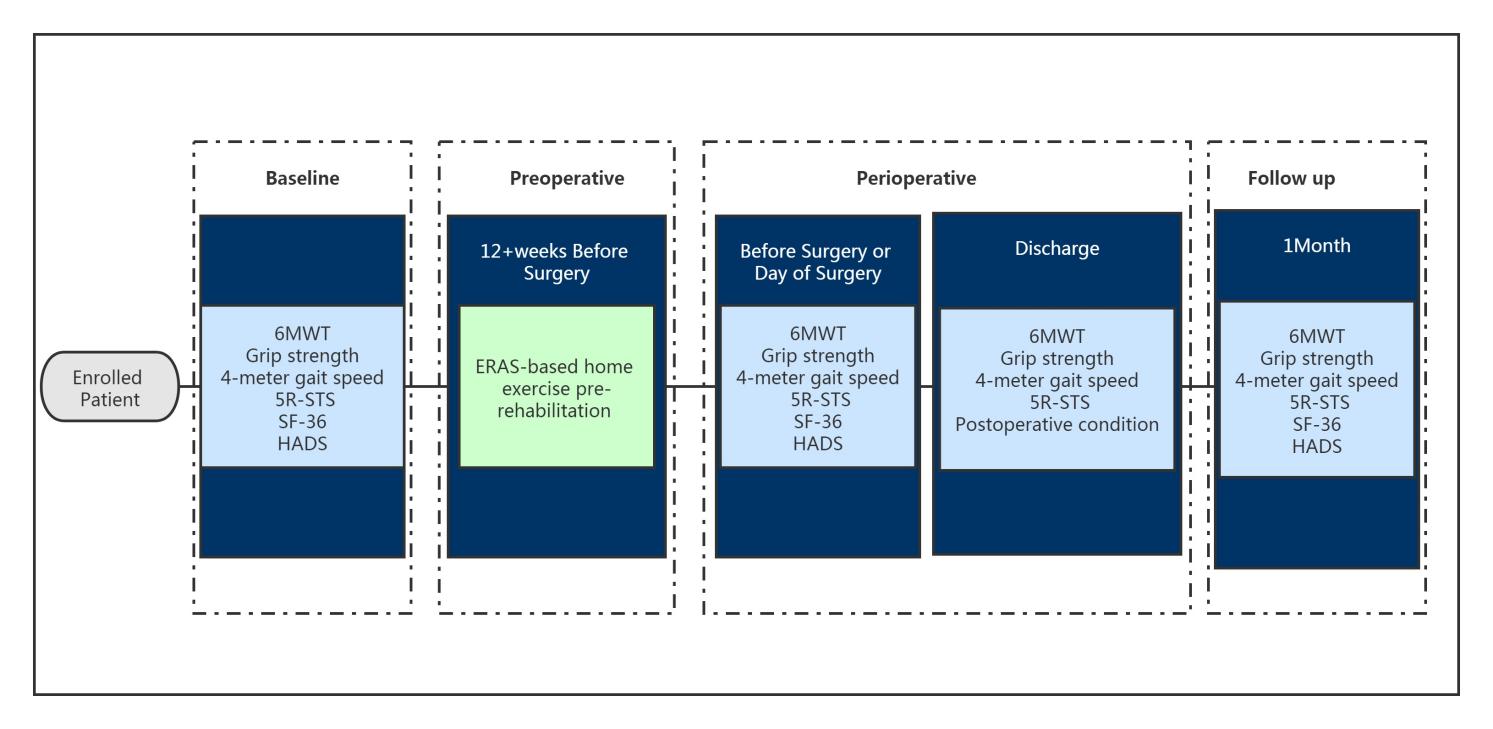


**Fig S1 Study schema**


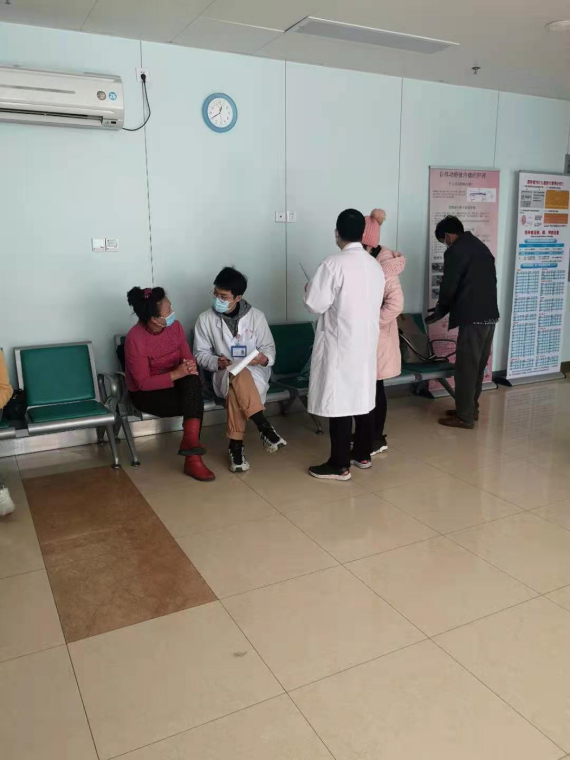

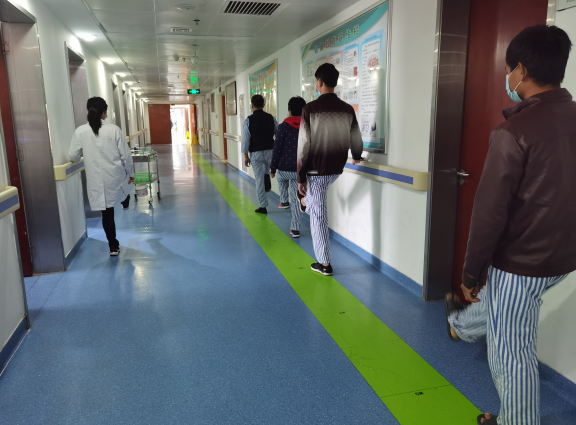


(a) (b)


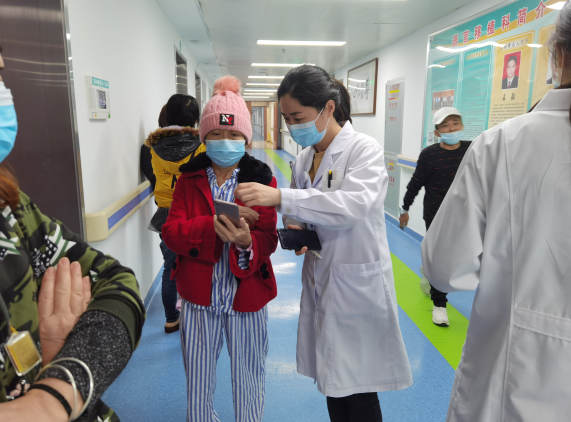


(c)

**Fig S2 pictures of the intervention (a):evaluation process (b):Face-to-face instruction in functional resistance exercise (c)Teaching the use of sports bracelet**


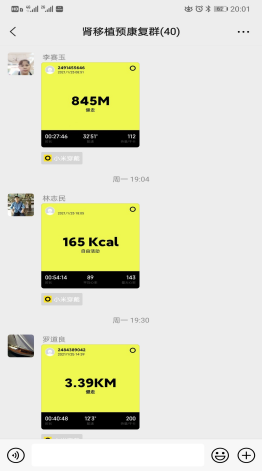


**Fig S3 Participants check-in exercise record in WeChat group**
